# White matter hyperintensities are an independent predictor of cognitive decline 3 years following first-ever stroke—results from the PROSCIS-B study

**DOI:** 10.1007/s00415-022-11481-5

**Published:** 2022-12-06

**Authors:** Huma Fatima Ali, Lea Fast, Ahmed Khalil, Eberhard Siebert, Thomas Liman, Matthias Endres, Kersten Villringer, Anna Kufner

**Affiliations:** 1 Berlin School of Mind and Brain, Humboldt-Universität zu Berlin, Berlin, Germany; 2 Klinik für Psychiatrie and Psychotherapie, Charité, Universitätsmedizin Berlin, Berlin, Germany; 3 Center for Stroke Research Berlin (CSB), Klinik Für Neurologie, Charité, Universitätsmedizin Berlin, Charitéplatz 1, 10117 Berlin, Germany; 4 Berlin Institute of Health (BIH), Berlin, Germany; 5 Max Planck Institute for Human Cognitive and Brain Sciences, Berlin, Germany; 6 Department of Neuroradiology, University Hospital of Berlin (Charité), Berlin, Germany; 7 Klinik und Hochschulambulanz für Neurologie mit Experimenteller Neurologie, Charité-Universitätsmedizin Berlin, Berlin, Germany; 8 German Centre for Cardiovascular Research (DZHK), Partner Site Berlin, Berlin, Germany; 9 German Center for Neurodegerenative Diseases (DZNE), Partner Site Berlin, Berlin, Germany; 10 ExcellenceCluster NeuroCure, Charité-Universitätsmedizin Berlin, Berlin, Germany

**Keywords:** White matter hyperintensities, Cerebrovascular risk factors, Cognitive impairment, Recurrent cerebrovascular events, First-ever ischemic stroke, Depression

## Abstract

**Background:**

White matter hyperintensities (WMH) are the result of cerebral small vessel disease and may increase the risk of cognitive impairment (CI), recurrent stroke, and depression. We aimed to explore the association between selected cerebrovascular risk factors (CVRF) and WMH load as well as the effect of increased WMH burden on recurrent vascular events, CI, and depression in first-ever ischemic stroke patients.

**Methods:**

431 from the PROSpective Cohort with Incident Stroke (PROSCIS) were included; Age-Related White Matter Changes (ARWMC) score was used to assess WMH burden on FLAIR. The presence of CVRF (defined via blood pressure, body-mass-index, and serological markers of kidney dysfunction, diabetes mellitus, and hyperlipoproteinemia) was categorized into normal, borderline, and pathological profiles based on commonly used clinical definitions. The primary outcomes included recurrent vascular events (combined endpoint of recurrent stroke, myocardial infarction and/or death), CI 3 years post-stroke, and depression 1-year post-stroke.

**Results:**

There was no clear association between CVRF profiles and WMH burden. High WMH lesion load (ARWMC score ≥ 10) was found to be associated with CI (adjusted OR 1.05 [95% CI 1.00–1.11]; *p* < 0.02) in a mixed-model analysis. Kaplan–Meier survival analysis showed a visible increase in the risk of recurrent vascular events following stroke; however, after adjustment, the risk was non-significant (HR 1.5 [95% CI 0.76–3]; *p* = 0.18). WMH burden was not associated with depression 1-year post stroke (adjusted OR 0.72 [95% CI 0.31–1.64]; *p* = 0.44).

**Conclusion:**

Higher WMH burden was associated with a significant decline in cognition 3 years post-stroke in this cohort of first-ever stroke patients.

**Supplementary Information:**

The online version contains supplementary material available at 10.1007/s00415-022-11481-5.

## Introduction

Cerebral small vessel disease (CSVD) is one of the most prevalent pathologies that neurologists and radiologists encounter in routine clinical practice. Not only does the presence of CSVD substantially increase the risk of stroke, but it is known to contribute to cognitive decline and dementia [1–3]. The increased availability of magnetic resonance imaging (MRI) in the clinical routine, has substantially increased the detection rates of CSVD; especially as an incidental finding in asymptomatic cases[2,4,5].

Imaging biomarkers of CSVD include small subcortical infarcts, lacunes, white matter hyperintensities (WMH), enlarged perivascular spaces, cerebral microbleeds, and global cerebral atrophy [4]. The presence of WMH are the most common imaging biomarker of CSVD, and are most easily detected on so-called fluid-attenuated inversion recovery (FLAIR) sequences. Although possible underlying pathologies behind WMH are heterogenous, histological studies suggest that WMH are most likely a result of chronic ischemia leading to demyelination and ultimately axonal loss [6–8]. WMH are so prevalent, that nearly 80% of healthy 60-year-olds and approximately 95% of people aged 90 years or older were found to have developed WMH [9,10]. Within the past decades, numerous modifiable risk factors for the development of WMH have been identified, including smoking, diabetes mellitus, arterial hypertension, hyperlipidemia, chronic kidney disease, and the presence of metabolic syndrome [11–13]. While CSVD and ultimately the presence of WMH can remain asymptomatic and is often merely an incidental finding on routine MRI [2], previous studies suggest that progressive WMH likely leads to increased stroke risk and early cognitive decline depending on distribution and localization within the brai n[3].

Interestingly, recent studies suggest that selected cerebrovascular risk factors lead to different WMH distribution patterns within the brain [3]. For example, a cohort study found that patients with chronic hypo- and hypertension present with different patterns and distributions of WMH (periventricular versus deep white matter) on MRI, with a history of hypertension leading to increased periventricular WMH burden [14]. Moreover, smoking has been linked to low cerebral blood flow, leading to increased WMH volumes across all age groups [15]. Furthermore, a recent study reported a strong association between type 2 diabetes with increased whole-brain WMH volume [16]. Most recently, a large population-based study found that obesity and associated low-grade systemic inflammation led to primarily deep WMH burden and comparatively less periventricular WMH volumes [17]. In other words, the presence or absence of selected risk factors is likely to influence WMH distribution patterns and ultimately may contribute to recurrent stroke risk and risk of dementia.

As the individual risk of stroke recurrence varies considerably among patients [18], patient-specific risk stratification is of enormous clinical importance. This is the case especially in younger patients with first-ever stroke, as early detection and initiation of secondary prevention could subsequently reduce recurrent cerebrovascular events. Therefore, we set out to investigate the relationship between latent or 'borderline' risk profiles (e.g., glucose intolerance without manifest diabetes mellitus) of common and well-known cerebrovascular risk factors and WMH burden in a comprehensive cohort of first-ever stroke patients. More specifically, we aimed to investigate the association between serological markers of selected cerebrovascular risk factors (i.e., HbA1c for diabetes mellitus, low-density lipoprotein ([LDL] for hypolipoproteinemia, and glomerular filtration rate [GFR] for kidney function) as well as clinical parameters (body mass index [BMI] and blood pressure) with WMH lesion load using the Age-related white matter changes (ARWMC) score. Subsequently, we investigated whether high WMH burden (defined as an ARWMC score ≥ 10) was associated with increased recurrent vascular events (including recurrent stroke, MI, and/or death) and cognitive impairment 3 years and depression one year following the first-ever stroke in a prospectively collected cohort of ischemic stroke patients.

## Methods

### Data availability statement

The data that supports the findings of this study are available upon reasonable request from the corresponding author [AK]. Raw imaging data are not publicly available as they contain information that could compromise patient privacy.

### Cohort characteristics

This is a retrospective analysis of the single-center prospective study *PROSpective Cohort with Incident Stroke (PROSCIS)* conducted at the Center for Stroke Research Berlin, Charité University Hospital. Patients aged ≥ 18 years with first-ever ischemic stroke were recruited between 2010 and 2013 after providing written informed consent. The exclusion criteria included any prior stroke, patients with a brain tumor or brain metastasis, and/or participation in an intervention study. A detailed description of the inclusion and exclusion criteria of the PROSCIS study can be found in the previously published study protocol [19]. The ethics committee approved the study for all recruiting centers in Berlin according to the Declaration of Helsinki and the study was registered in clinicaltrials.org (NTC01363856). For this study, patients with at least one MRI during standard clinical care following the index event (within 7 days following stroke onset) were included in the analysis.

### MRI assessment

The MRI protocol included the following sequences: T2*, diffusion-weighted imaging (DWI), and fluid-attenuated inversion recovery (FLAIR) images. All MRI examinations were conducted on a 3.0 Tesla or 1.5 Tesla Siemens MRI scanner. Post-processing of MRI images was performed offline with MRIcron Software from the Center for Advanced Brain Imaging (University of South Carolina, Chris Rordan, USA).

The ARWMC visual rating scale developed by Wahlund and team [20] was used to rate WMH on FLAIR images of all patients included in this study. Rating was performed independently by two evaluators (neurologist A.K. and senior neuroradiologist K.V.). The ARWMC score ranges from 0 to 30 with an assessment of both sides of the brain and pre-specified regions of the brain, which includes frontal, parieto-occipital, temporal, basal ganglia, and infra-tentorial. Each region’s grading ranges from 0 to 3 where grade 0 is occasional or non-punctate WMH; grade 1, multiple punctate WMH; grade 2, bridging of punctate WMH into confluent lesions; and grade 3, widespread confluent WMH [20]. For this study, ARWMC score was dichotomized with a cut-off of < 10 representing a lower WMH load and ≥ 10 as a higher WMH load [21].

### Clinical assessment

All patients included in the study were examined and interviewed within 7 days after the onset of stroke symptoms. Clinical parameters included basic patient demographics, education status, smoking status, history of diabetes mellitus, hypertension, atrial fibrillation, and hyperlipoproteinemia. Clinical parameters documented on admission (BMI and systolic and diastolic blood pressure) as well as serological parameters (HbA1c, LDL, and GFR) were used in this analysis.

Clinical examinations included stroke severity and functional outcome on admission. Stroke severity was assessed using the National Institutes of Health Stroke Scale (NIHSS), a 15-item scale ranging from 0 to 42, with higher values reflecting a more severe neurological deficit^[[[22]]]^. Functional outcome was assessed using the modified Rankin Scale (mRS) score, ranging from 0 to 6. mRS is a measure of disability where 0 reflects no symptoms; 5, severe disability leading to nursing aid; and 6, death^[[[23]]]^.

### Cerebrovascular risk factors assessment

Diabetic status was evaluated by analyzing the HbA1c levels, which were categorized into normal as 4.5–5.7%, borderline as 5.8–6.4%, and pathological as ≥ 6.5% [24]. LDL was categorized into normal as ≤ 70 mg/dL; borderline as 71–139 mg/dL; and pathological as ≥ 140 mg/dL. BMI was divided into normal as ≤ 25, borderline as 26–29, and pathological as ≥ 30 [25]. Blood pressure readings were analyzed separately for systolic and diastolic readings. The mean of three separate blood pressure measurements on admission was calculated and then categorized into the following profiles: systolic blood pressure was categorized into hypotension (≤ 120 mmHg), normal (121–139 mmHg) and hypertension (≥ 140 mmHg). Similarly, diastolic blood pressure readings were categorized into hypotension (≤ 80 mmHg), normal (81–89 mmHg), and hypertension (≥ 90 mmHg) [26]. Kidney function was analyzed using the estimated glomerular filtration rate (eGFR), which was categorized into normal as ≥ 90 mL/min; borderline as 61 mL/min to 89 mL/min; and pathological as ≤ 60 mL/min [27]. The definitions and cut-offs of the selected cerebrovascular risk factors included in this analysis are summarized in Supplementary Table 1.

### Primary endpoints

All three primary endpoints were assessed at 1-, 2-, and 3 years post-stroke.

#### Cognitive assessment

Cognitive status was evaluated at baseline using the Mini-Mental State Examination (MMSE) [28]. During follow-up, cognitive status was assessed using the Modified Telephone Interview for Cognitive Status (TICS-M), which is a modified variant of the MMSE which has been validated for use via telephone interviews [29,30]. The MMSE is a 30-point questionnaire for cognitive impairment to screen for dementia where a cut-off of ≥ 24 indicates normal cognition and ≤ 23 indicates cognitive impairment. The TICS-M is an 11-test-item questionnaire yielding a total score of 50 points where a cut-off of ≤ 31 represents cognitive impairment and ≥ 32 represents normal cognition.

#### Depression assessment

Depression status was assessed using the Center for Epidemiologic Studies Depression Scale (CES-D) [31]. Depression is an inherently difficult endpoint to assess as it can fluctuate for individual patients over time and is likely influenced by many factors including mobility status, stroke severity etc. For this reason, depression scores at only 1-year follow-up were used in the analysis. CES-D is a 20-item questionnaire comprising six scales that reflect significant facets of depression based on self-reported information on depressive mood, feelings of guilt and worthlessness, helplessness and hopelessness, psychomotor retardation, loss of appetite, and sleep disturbance. The cut-off value of ≥ 16 represents clinical depression [32]. To account for missing scores we applied inverse probability-weighted estimation of death as part of a sensitivity analysis.

#### Combined endpoint of recurrent vascular events

The combined endpoint of recurrent vascular events included the first of either recurrent stroke, myocardial infarction (MI), or death by any cause. Incidences of recurrent vascular events were assessed using Rose Angina Questionnaire for cardiovascular events and Stroke Symptom Questionnaire for cerebrovascular events [33,34]. Requested measures were collected at baseline and follow-up of 1-, 2-, and 3 years post-stroke. The follow-up was carried out via a structured telephonic questionnaire or mail. In case of any positive vascular outcome event, the information was validated by the admitting hospital or the treating physician.

### Statistical analysis

Baseline characteristics of categorical data is represented in absolute and relative frequencies, and the distribution of continuous data is described as median and interquartile ranges (IQR). The selection of covariates for our analyses was evaluated by Directed Acyclic Graphs (DAGs). DAGs, a derivate of causal diagrams in epidemiology, provide a graphical representation of rigorous mathematical methodology for mapping all a priori assumptions surrounding a causal question [35]. In our study, a separate DAG was created for each causal relationship of interest to identify the potential variables of confounding while avoiding colliders and intermediate pathways using the online tool, Dagitty [http://dagitty.net/] [36]. All DAGs used to inform our analytic strategy have been uploaded onto an open repository and can be accessed via the following link: [https://doi.org/10.6084/m9.figshare.20152487].

Logistic regression (unadjusted and adjusted analysis) was performed to assess the association of borderline vs. pathological cerebrovascular risk factors with dichotomized ARWMC score; interactions between CVRF categories were not considered in the analysis. The models were adjusted based on DAG graphs created for each variable as described above. The model for HbA1c was adjusted for age, sex, smoking, BMI, and hyperlipidemia; models for systolic and diastolic blood pressures were adjusted for age, sex, diabetes, smoking, BMI, and hyperlipidemia; LDL model was adjusted for age, sex, BMI, and smoking; BMI model for age, sex, and smoking; and GFR model for age, sex, diabetes, BMI, hypertension, hyperlipidemia, smoking, and atrial fibrillation.

Kaplan–Meier survival analysis was performed to analyze the association of WMH lesion load (ARWMC score ≥ 10) on the recurrent vascular events within 3 years post-stroke. We report unadjusted and adjusted hazard ratios (HR) with 95% CI obtained from Cox proportional hazards model, and adjustment was made for age, sex, and hypertension.

The effect of WMH lesion load on cognition status (dichotomized at ≤ 23) 3 years post-stroke was analyzed by (non-linear) mixed-model analysis followed by additional sensitivity analysis which excluded all deaths post-stroke within three years. Adjustments were made for age, sex, education status, smoking, hypertension, diabetes, atrial fibrillation, and hyperlipidemia. All models included subject ID as random effects and WMH and cognition scores as fixed effects.

Depression at 1-year post-stroke relative to WMH severity was analyzed by a logistic regression model followed by weighted analysis for probability of death. An adjustment was made for age, sex, CI at baseline, NIHSS, and mRS status at 1-year post-stroke.

For all models, a 2-sided *p*-value < 0.05 was considered statistically significant. All analyses were performed using the software STATA IC version 15 (StataCorp, College Station, Texas, USA).

## Results

### Cohort description

627 patients in total are included in PROSCIS, of which 431 patients presenting with first-ever ischemic stroke with an available MRI following the index event were analyzed. The mean age of this cohort was 66.8 years (standard deviation [SD] 13.2), of which 244 (38.9%) were females, with baseline NIHSS of (median NIHSS 2 [IQR 01–05]. The 196 patients who did not receive an MRI at the baseline had a mean age of 69.6 years [SD 12.42], and median baseline NIHSS of 3 (IQR 2–5). Baseline characteristics of the entire analyzed cohort are described in Table 1.

**Table 1:** Baseline clinical and imaging characteristics of the PROSCIS-B cohort.

Total patient cohort	
Demographics	
Age, mean (± SD)	66.9 (± 13.2)
Sex, female, *n* (%)	244 (38.9%)
Education level ≥ 10 years, *n* (%)	173 (28.8%)
Cardiovascular risk factors	
Hypertension, *n* (%)	409 (65.2%)
Diabetes, *n* (%)	138 (22%)
Hyperlipidemia, *n* (%)	128 (22.7%)
Atrial fibrillation, *n* (%)	135 (21.5%)
BMI, Mean (SD)	27.5 (4.9)
Current smoking, *n* (%)	173 (28.0%)
Stroke etiology	
Large artery atherosclerosis, *n* (%)	169 (26.9%)
Cardioembolic stroke, *n* (%)	149 (23.8%)
Small vessel occlusion, *n* (%)	96 (15.3%)
Other, *n* (%)	22 (3.5%)
Unknown, *n* (%)	191 (30.5%)
Baseline clinical characteristics	
NIHSS at admission, Median[IQR]	2 [1–5]
mRS at admission, Median [IQR]	2 [1–5]
Infarct pattern	
Territorial with subcortical and cortical, *n* (%)	110 (17.5%)
Subcortical, *n* (%)	112 (17.9%)
Scattered infarct, *n* (%)	118 (18.8%)
Lacunar, *n* (%)	2 (0.3%)
Infratentorial, *n* (%)	107 (17.1%)
Watershed, *n* (%)	33 (5.3%)
ARWMC score	
ARWMC score, Median [IQR]	5 [3–9]

*SD* standard deviation, *IQR* interquartile range, *BMI* body mass index, *NIHSS* National Institute of Health Stroke Scale, *mRS* modified Rankin Score, *ARWMC* Age-Related White Matter Changes

### Cerebrovascular risk factors and WMH

Logistic regression analysis found no significant association across cerebrovascular risk factor categories (normal, borderline, and pathological as defined in Supplementary Table 1), with ARWMC score ≥ 10 (Table 2). For a graphical depiction of ARWMC score distribution (as a continuous variable) across cerebrovascular risk factor categories, please refer to the violin plots depicted in Supplementary Fig. 1.

**Table 2:** Logistic regression analysis for effects of borderline and pathological clinical/serological risk profiles and white matter hyperintensities (ARWMC score ≥ 10)

	*n* (%)	Unadjusted			Adjusted		
		Odds ratio	95% Confidence interval	*p*-value	Odds ratio	95% Confidence interval	*p*-value
HbA1c^a^							
Normal	259 (43.9%)	Ref					
Borderline	152 (25.8%)	1.50	0.86–2.56	0.15	0.99	0.52–1.91	0.99
Pathological	179 (30.3%)	1.21	0.69–2.09	0.49	0.93	0.49–1.78	0.84
Systolic BP^b^							
Normal	116 (19.3%)	Ref					
Borderline	205 (34.1%)	0.81	0.39–1.66	0.57	0.57	0.25–1.28	0.17
Pathological	280 (46.6%)	1.44	0.75–2.76	0.26	1.03	0.49–2.16	0.93
Diastolic BP^b^							
Normal	398 (66.1%)	Ref					
Borderline	99 (16.4%)	1.07	0.58–1.95	0.82	1.21	0.58–2.49	0.60
Pathological	105 (17.4%)	0.91	0.49–1.71	0.78	1.57	0.74–3.29	0.23
LDL^c^							
Normal	49 (8.2%)	Ref					
Borderline	362 (61.2%)	0.83	0.35–1.96	0.67	1.60	0.55–4.60	0.38
Pathological	180 (30.5%)	1.05	0.43–2.56	0.91	2.25	0.75–6.68	0.14
BMI^d^							
Normal	261 (42.3%)	Ref					
Borderline	206 (33.4%)	0.96	0.58–1.60	0.88	1.00	0.57–1.75	0.99
Pathological	150 (24.3%)	0.74	0.40–1.35	0.33	0.99	0.51–1.90	0.98
eGFR^e^							
Normal	193 (32.2%)	Ref					
Borderline	282 (47.1%)	2.35	1.32–4.18	0.00	0.91	0.43–1.92	0.79
Pathological	124 (20.7%)	3.32	1.66–6.64	0.00	0.91	0.35–2.36	0.84

*BP* blood pressure, *LDL* low-density lipoprotein, *BMI* body mass index, *GFR* glomerular filtration rate

^a^Adjusted for- age, sex, smoking, BMI, hyperlipidemia

^b^Adjusted for- age, sex, diabetes, smoking, BMI, hyperlipidemia

^c^Adjusted for- age, sex, BMI; smoking

^d^Adjusted for- age, sex, smoking

^e^Adjusted for- age, sex, diabetes, BMI, hypertension, hyperlipidemia, smoking, atrial fibrillation

Pathological HbA1c values had an odds ratio (OR) of 1.21; (95% CI 0.69–2.09) for high WMH load. Pathological diastolic blood pressure values had an adjusted OR of 1.57 (95% CI 0.74–3.29). Neither systolic blood pressure, high LDL, BMI, nor chronic kidney disease defined by GFR showed a significant association with increased WMH load (Table 2). Due to the fact that systolic and diastolic blood pressure values may vary substantially at the time of acute stroke (irrespective as to whether a true diagnosis of hypertension exists), we performed an additional logistic regression analysis including the history of hypertension (yes/no); here we found a significant association with WMH load and history of hypertension with an adjusted OR of 2.44 (95% CI 1.30–4.57; *p* = 0.00).

For a visual depiction of the results of the regression analysis (unadjusted and adjusted OR with 95% CI) for all cerebrovascular risk factors and ARWMC score, please refer to Supplementary Fig. 2.

### WMH and recurrent vascular events

**Fig. 1:**
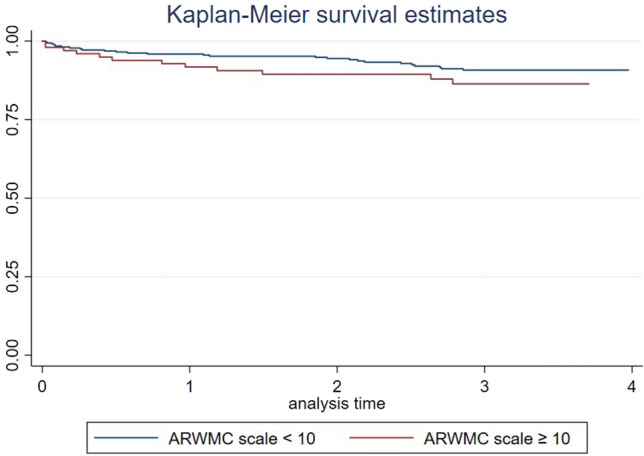
Kaplan–Meier survival analysis curve showing association of white matter hyperintensities with the incidence of recurrent stroke, myocardial infarction, and/or death on follow-up of 1-, 2, and 3-years post-stroke

In the Kaplan–Meier survival analysis for the combined vascular endpoint (recurrent stroke, MI, and/or death) patients with high ARWMC scores had visibly lower survival rates when analyzed 3 years post-stroke (Fig. 1). In the cox-regression analysis, patients with a ARWMC score ≥ 10 had an unadjusted HR of 1.6 (95% CI 0.79–3.13; *p* = 0.20) and adjusted HR of 1.5 (95% CI 0.76–3.03; *p* = 0.18) for the combined vascular endpoint 3 years post-stroke.

### WMH and cognitive impairment

A significant association between higher WMH load and cognitive impairment 3 years post-stroke was found in the mixed model analysis with an unadjusted OR of 2.22 (95% CI 1.20–4.08; *p* = 0.01) and an adjusted OR of 1.05 (95% CI 1.00–1.11; *p* = 0.02). The sensitivity analysis produced similar results. The models were adjusted for age, sex, education status, smoking, hypertension, diabetes, atrial fibrillation, and hyperlipidemia (Table 3).

**Table 3:** Mixed model analysis showing the effect of white matter hyperintensities burden with long-term cognitive function

ARWMC Score	Unadjusted OR	[95% CI]	*p*-value	Adjusted OR^a^	[95% CI]	*p*-value
Cognitive impairment	2.22	1.21–4.08	0.01	1.05	1.00–1.11	0.02
Sensitivity analysis model						
Cognitive impairment	1.88	0.98–3.56	0.05	1.05	1.00–1.11	0.03

^a^Adjusted for age, sex, education status, smoking, hypertension, diabetes, atrial fibrillation, and hyperlipidemia

### WMH and depression

In logistic regression analysis, high WMH load had an adjusted OR of 0.83 (95% CI 0.39–1.77; *p* = 0.64), following adjustment for age, sex, cognitive impairment at baseline, NIHSS, and mRS at 1-year post-stroke. An additional inverse probability weighted analysis also showed no significant association between increased WMH load and depression 1-year post-stroke with an adjusted OR of 0.72 (95% CI 0.31–1.64; *p* = 0.44).

## Discussion

The main finding of this study is that increased WMH load (defined as ARWMC score ≥ 10) was significantly associated with cognitive impairment after 3 years in first-ever stroke patients. In addition, a higher WMH load at the time of stroke is likely associated with recurrent vascular events including recurrent stroke, MI, and/or death in patients with first-ever ischemic stroke. One of the primary aims of the current study was to assess whether selected cardiovascular risk profiles affect the WMH burden in first-ever stroke patients. While a history of hypertension was significantly associated with increased WHH load, the current study found no apparent differences in WMH load across so-called borderline and pathological cerebrovascular risk profiles assessed via clinical and serological markers on admission.

Similar to previous studies, we found that a history of arterial hypertension is associated with an increased burden of WMH [9,14]. However, we found no association between baseline systolic or diastolic blood pressure values and increased WMH load in the current analysis. A previously published meta-analysis of four trials has shown that anti-hypertensive medication could significantly slow the progression of WMH [37]. Unfortunately, in the current study, medication status was not available and could not be considered. This may have affected our categorizations and ultimately our results. For example, patients with a long-term history of hypertension and a recent start of anti-hypertensive medication may have normotensive systolic blood pressure at the time of the index event. The same might be true for serological markers of hypercholesterolemia and diabetes mellitus with LDL cholesterol and HbA1c, respectively. An earlier study found a significant association of diabetes mellitus with increased WMH severity, particularly in terms of increased whole-brain WMH volume in a population-based sample of 99 patients with diagnosed diabetes mellitus type 2 [16]. These patients had an average of nearly 9 years of diabetes duration, therefore generalizability of these findings to a cohort of first-ever stroke patients where diagnostic work-up following stroke often leads to first diagnosis of cerebrovascular risk factors is limited. Although previous population-based studies also suggest high BMI to be significantly associated with the development of increased deep WMH lesions [17], here we also failed to find a clear association between high BMI and WMH burden.

While arterial hypertension, diabetes mellitus, hyperlipoproteinemia, obesity, and poor kidney function are all well-known cerebrovascular risk factors and likely do play an important role in the development of WMH [11,14–18,38,39], we did not see relevant associations with our selected risk profiles and higher ARWMC scores in this cohort of first-ever stroke patients. It is important to note that the aforementioned studies are all population-based studies; in other words, patients were selected based on the presence of the risk factor of interest. Therefore, generalizability to a cohort of first-ever stroke patients is likely limited. Furthermore, the current cohort has a relatively mild WMH burden (median ARWMC score of 5 [IQR 3–9]). The visual assessment of WMH lesion load via the ARWMC score may not be sensitive enough to detect associations of risk factors and very early manifestations of ARWMC in first-ever stroke patients. An analysis using a quantitative assessment of WMH volume is certainly warranted to explore this topic further.

The Kaplan–Meier survival analysis for recurrent vascular events showed a visible increase in risk in patients with ARWMC score ≥ 10 across 3 years following stroke (Fig. 1); however, these results were not statistically significant in adjusted cox-regression analysis (HR 1.52 95% CI 0.76–3.03; *p* = 0.18). This may be due to the small sample size in the current study. Literature has shown that the risk of stroke recurrence varies considerably among patients and depends on age, sex, and the presence of co-morbidities [40,41]. In line with our observation, a recent Danish Stroke Registry-based observational study including 832 patients reported a significant association between WMH and increased risk of recurrent stroke (HR 5.28; 95% CI [1.98–14.07]) [42] in a cohort of incident ischemic stroke patients.

We found a significant association of increased WMH lesion load with CI 3 years following first-ever stroke. Additional sensitivity analysis also yielded a similar result (adjusted OR of 1.1 95% CI 1.0–1.1; *p* = 0.03). Previous studies on the effect of WMH on cognitive decline have yielded somewhat controversial results; while several studies found no clear association between WMH and cognitive decline [43,44], others have found an increase in WMH volume to be an independent predictor of CI in both stroke cohorts as well as population-based studies [18,45]. Recently, a study reported that increased WMH volume was associated with cognitive decline in patients < 80 years of age following a minor stroke or TIA [44]. Since WMH are very commonly seen on MRI even in healthy subjects, the interpretation of these as a proxy for risk of CI can be complex and should still be reported and applied with caution. However, there is increasing evidence for a causal relationship between increased WMH load and the risk of the development of cognitive decline which could have important clinical implications i.e., early identification of an increased risk of CI could play a major role in initiating secondary prevention strategies in patients at risk.

We found no association between high WMH load and depression post-stroke in this analysis. There are conflicting results reported in the literature on the effect of WMHs on the development of depression. Although several population-based studies have found that WMH progression is associated with depression and antidepressants can even slow WMH progression [46], the effects of WMH burden in stroke patients on post-stroke depression remains unclear. As of yet, different WMH patterns such as peri-ventricular WMH and deep WMH are found to affect depression at baseline or a yearlong follow-up in stroke patients, respectively [47]. Depression is an inherently difficult endpoint to assess in stroke patients, as depression can fluctuate over time for an individual patient and is affected by many factors (medication, functional status post-stroke, and co-morbidities), for which we could not account for in the current analysis. Nonetheless, very few studies have explored the association between WMH and depression in stroke patients, and we recommend more comprehensive studies in independent cohorts to further explore this interesting and clinically highly relevant topic.

To the best of our knowledge, this is the first study to analyze the association of selected clinical and serological cardiovascular risk profiles (borderline vs. pathological) with WMH burden in a comprehensive cohort of first-ever stroke patients. However, our study has several limitations that warrant discussion. First and foremost, this is a retrospective, exploratory analysis of a prospective cohort that was not designed to address our primary research questions, therefore we did not adjust for multiple testing. Furthermore, data on patients’ pre-stroke medication was not available for this cohort, therefore we could not account for the possible influence of pre-stroke medications on baseline clinical and serological risk profiles like blood pressure or HbA1c. This may partially explain why we failed to see a clear association between selected risk profiles and WMH burden. Similarly, we cannot exclude the possible presence of pre-stroke cognitive impairment in this cohort; the use of the mixed-model analysis for cognitive impairment in this analysis only partially compensates for this limitation. Additionally, we could only include patients within PROSCIS that received an MRI, which introduces a potential bias into our cohort because patients with contraindications for an MRI were automatically excluded from the current analyses. This may be reflected by the relatively mild strokes of the patients included in our analysis (median NIHSS at the admission of 2 [IQR 1–5]). Therefore, generalizability to more severely affected stroke cohorts is limited.

A fundamental limitation of this study is that we applied a qualitative visual method for assessing WMH burden (i.e., the ARWMC score). This did not allow us to determine the WMH burden in more detail, i.e., total WMH volume or anatomical distribution (peri-ventricular versus deep white matter). ARWMC score may not be sensitive enough to detect very early manifestations of ARWMC and a ceiling effect at higher scores is to be expected. However, the ARWMC score is a well-known and well-established scoring system for assessing WMH burden in the clinical setting. The advantage of applying the ARWMC score as done in this study is that it increases the clinical applicability of the results. Nonetheless, a more detailed analysis of WMH distribution pattern and volume in terms of cerebrovascular risk profiles and long-term outcomes is undoubtedly warranted to increase our understanding of the prognostic value of early WMH progression. Finally, we can not rule out entirely the possibility that some patients included in the current analysis had other less common causes of WMH – for example, amyloid angiopathy, previous radiation exposure, or genetic disorders. The further assessment of additional imaging biomarkers of CSVD (lacunes, microbleeds, cerebral atrophy, and enlarged perivascular spaces) was beyond the scope of the current study but would also be essential to fully comprehend the causal effect of CSVD on recurrent vascular events, cognitive decline, and depression in first-ever stroke patients with CSVD.

## Conclusion

In summary, our study found no clear associations between selected cerebrovascular risk profiles and WMH burden; this may be due to the small sample size and the methodology applied (i.e., ARWMC score vs. quantitative assessment of WMH including volume and anatomical distribution). However, our study supports the hypothesis that WMH burden leads to a significantly higher risk of cognitive decline and possibly even recurrent vascular events following the first-ever stroke which may allow us to identify patients at risk and initiate early patient-individualized preventative treatment strategies if validated in further studies.

## Supplementary Information

Below is the link to the electronic supplementary material.

Supplementary file1 (DOCX 156 KB)
