# Supplementary material for: White matter hyperintensities are an independent predictor of cognitive decline 3 years following first-ever stroke—results from the PROSCIS-B study
Source: J Neurol. 2022 Dec 6;270(3):1637–46. doi: 10.1007/s00415-022-11481-5 (PMC9971076; doi:10.1007/s00415-022-11481-5)
Supplement: Supplementary file 1 — Supplementary file1 (DOCX 156 KB) [file 415_2022_11481_MOESM1_ESM.docx]

**SUPPLEMENTARY DATA**

**White matter hyperintensities are an independent predictor of cognitive decline three years following first-ever stroke - a PROSCIS-B study**

Huma Fatima Ali, Lea Fast, Ahmed Khalil, Eberhard Siebert, Thomas Liman, Matthias Endres, Kersten Villringer***,** Anna Kufner*

^1^ Berlin School of Mind and Brain, Humboldt-Universität zu Berlin, Berlin, Germany

^2^Klinik für Psychiatrie and Psychotherapie, Charité – Universitätsmedizin Berlin, Berlin, Germany

^3^Center for Stroke Research Berlin (CSB), Charité – Universitätsmedizin Berlin, Berlin, Germany

^4^Berlin Institute of Health (BIH), Berlin, Germany

Max Planck Institute for Human Cognitive and Brain

Sciences, Berlin, Germany

Max Planck Institute for Human Cognitive and Brain

Sciences, Berlin, Germany

Max Planck Institute for Human Cognitive and Brain

Sciences, Berlin, Germany

Max Planck Institute for Human Cognitive and Brain

Sciences, Berlin, Germany

Max Planck Institute for Human Cognitive and Brain

Sciences, Berlin, Germany

^5^Max Planck Institute for Human Cognitive and Brain Sciences, Berlin, Germany.

^6^Department of Neuroradiology, University Hospital of Berlin (Charité), Berlin, Germany

^7^Klinik und Hochschulambulanz für Neurologie mit Experimenteller Neurologie, Charité-Universitätsmedizin Berlin, Germany

^8^German Centre for Cardiovascular Research (DZHK), partner site Berlin, Germany

^9^German Center for Neurodegerenative Diseases (DZNE), partner site Berlin, Germany

^10^ExcellenceCluster NeuroCure, Charité-Universitätsmedizin Berlin, Germany

**Corresponding author:**

Anna Kufner, MD, PhD

Center for Stroke Research Berlin (CSB)

Klinik für Neurologie

Charité–Universitätsmedizin Berlin

Charitéplatz 1, Berlin 10117, Germany

E-mail: anna.kufner@charite.de

Tel: 030 450 560 137

| **Panel: Categorization of cardiovascular risk factors** | | | |
| --- | --- | --- | --- |
| CVRF | Normal | Borderline | Pathological |
| HbA1c | 4.5 – 5.7 | 5.8 – 6.4 | ≥ 6.5 |
| GFR (mL/min | ≥ 90 | 61 – 89 | ≤ 60 |
| LDL (mg/dL) | ≤ 70 | 71 – 139 | ≥ 140 |
| BMI | ≤ 25 | 26 – 29 | ≥ 30 |
| Blood Pressure | Hypotension | Normal | Hypertension |
| Systolic blood pressure (mmHg) | ≤120 | 121 to 139 | ≥140 |
| Diastolic blood pressure (mmHg) | ≤80 | 81 to 89 | ≥90 |

**Supplementary Table 1:** Summary of definitions and cut-off values applies to the selected cerebrovascular risk factors (CVRF)

**Supplementary Figure legends**


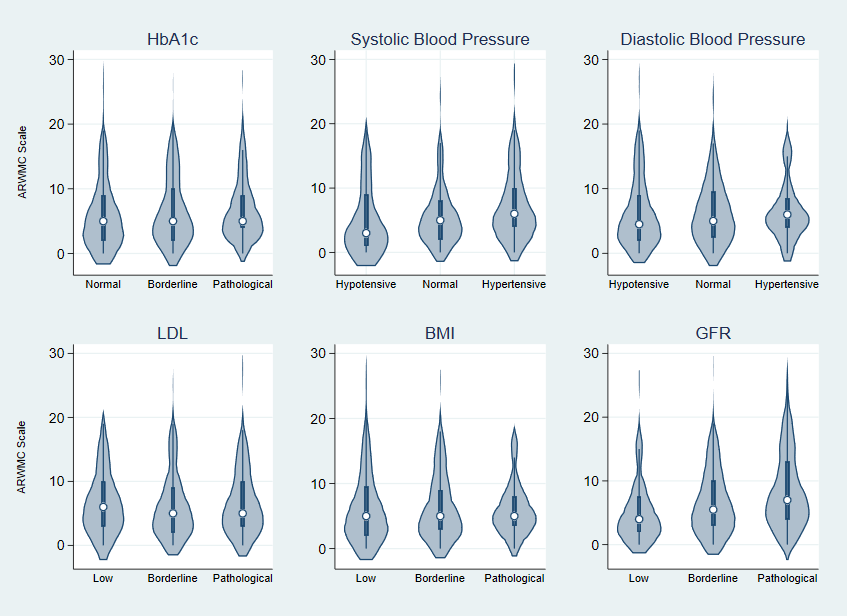


**Supplementary Figure 1** Violin plots for normal, borderline and pathological clinical/serological risk profiles of selected cerebrovascular risk factors.

ARWMC = Age related white matter changes, LDL = low-density lipoprotein; BMI = body mass index; GFR = glomerular filtration rate.


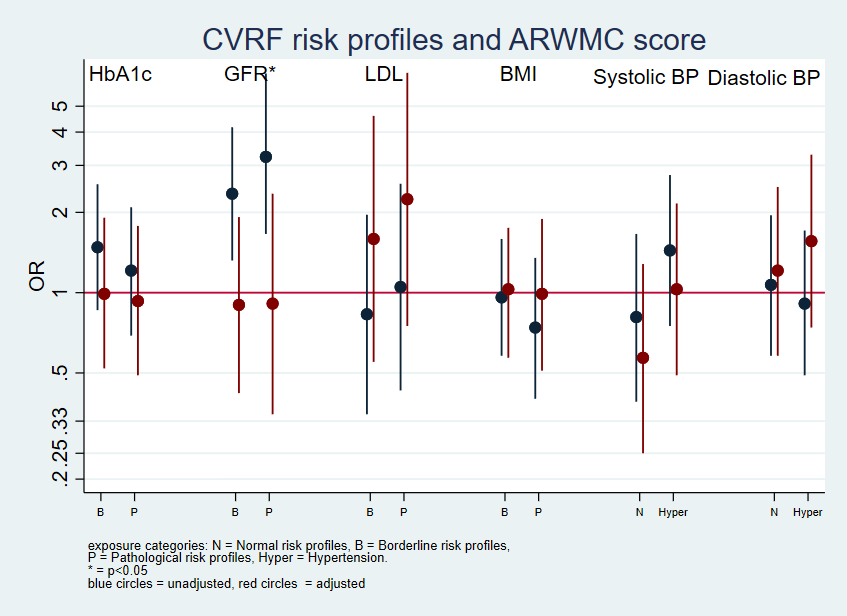


**Supplementary Figure 2** Regression analysis diagram for cardiovascular risk factors and WMH.

ARWMC = Age related white matter changes, GFR = Glomerular filtration rate; LDL = Low-density lipoprotein; BMI = Body mass index; BP = blood pressure.

Adjustment sets:

HbA1c = age, sex, smoking, BMI, and hyperlipidemia.

Systolic and diastolic blood pressure = age, sex, diabetes, smoking, BMI, and hyperlipidemia.

LDL = age, sex, BMI, and smoking.

BMI = age, sex, and smoking.

GFR = age, sex, diabetes, BMI, hypertension, hyperlipidemia, smoking, and atrial fibrillation.
